# What We Learned from Performing the Inverse Malek Procedure to Repair Bilateral Cleft Lips and Palates: A Single-Center Retrospective Study

**DOI:** 10.3390/jcm13071939

**Published:** 2024-03-27

**Authors:** Karim Al-Dourobi, Tessa Mermod, Marie-Thérèse Doan, Georges Herzog, Martin Broome, Oumama El Ezzi, Anthony de Buys Roessingh

**Affiliations:** 1 Plastic, Reconstructive and Hand Surgery Service, Lausanne University Hospital, University of Lausanne, CH-1011 Lausanne, Switzerland; karim.al-dourobi@chuv.ch (K.A.-D.);; 2 Children and Adolescent Surgery Department, Multidisciplinary Consultations of Facial Cleft, Lausanne University Hospital, University of Lausanne, CH-1011 Lausanne, Switzerlandgeorges.herzog@me.com (G.H.);

**Keywords:** bilateral cleft, Malek, oronasal fistula, pharyngeal flap, speech

## Abstract

**Background**: This study reviews the surgical and functional outcomes of children diagnosed with a bilateral cleft lip and palate and treated by the same surgical team following specific surgical protocols 18 years after surgery and during the follow-up. **Methods**: Based on a single-center retrospective design, demographic and surgical data were gathered by the authors from international institutions. Most of the data were quantitative in nature, and descriptive statistical and non-parametric tests were employed for analysis. All children born with a bilateral cleft from 1982 to 2002 were considered. Children affected by a syndrome were excluded. Complications and speech results were the main items measured. **Results:** Thirty patients were selected; 73.3% were treated using the inverse Malek procedure, and 26.7% underwent a modified two-stage procedure. Seventy percent developed an oronasal fistula. An alveolar bone graft was performed in 83%, and 53.3% underwent Le Fort osteotomy. Thirty-six percent required a pharyngeal flap, with good speech results. The median number of times general anesthesia was used among all the interventions considered was 5.5 (4.25–6). **Conclusions:** This study presents the long-term results of using the inverse Malek procedure to treat children with a bilateral cleft lip and palate. It is shown that this is related to a high risk of developing a fistula, but has good long-term speech results.

## 1. Introduction

Orofacial clefts (OFCs) are common congenital malformations [1]. Their frequency ranges from 1/700 to 1/2500 births, depending on the person’s geographic origin and racial background [1,2]. Thirty percent of OFCs are part of a more general syndrome, and more than 300 syndromes have been identified as playing a direct part in the forming of an OFC [1,2]. The majority of OFCs (70%) are not part of a syndrome and can be either isolated or related to other malformations [3]. These clefts result from complex interactions between genetic and environmental factors [4,5,6,7]. The OFC phenotypes commonly accepted are a cleft lip and palate (CLP), an isolated uni- or bilateral cleft lip (CL), and an isolated cleft palate (CP). A unilateral cleft lip and palate (UCLP) is the most frequent presentation, accounting for approximately for 30–50% of cases [2,8]. Isolated CLs or CPs account for 20–25%, respectively. Bilateral cleft lip and palates (BCLPs) are the most uncommon presentation of clefts (10%) and the most complicated and challenging to repair [9,10,11]. The main problems to be resolved are the protrusion of the premaxilla (incisive bone) that increases the transvers width of the cleft and the presence of the prolabium, which lacks muscle and is not vermillion, and the loss of the Cupid’s bow and of almost the entire philtrum [9]. These central upper lip features are very complex to restitute and virtually nonexistent in children with a BCLP [10,11].

For BCLP repair, lip closure can be performed in one or two stages. If it is performed in one single stage, with incisions on both sides, the extensive denuding of tissue may impair the vascular supply to the bone structure and the soft tissue, leading to necrosis. An abnormally short columella is one of the hallmarks of these malformations [10].

Mixed surgical models and timings have been widely adopted since 1970 [12]. The central point of discussion has been the sequence of the closure of the soft tissue and hard palate. If it is true that scars on the periosteum can induce growth abnormalities of the midface [12,13], delaying the closure of the palate (both hard and soft) will induce speech problems [14]. Studies have since shown that the timing of soft tissue and palatal closure is more important than their sequence [13,15,16,17,18], delaying hard palate closure results in speech impairment and shows no benefit for maxillofacial growth [14,19,20]. Regarding the soft palate, its early closure may reduce the width of the hard palate cleft, but does not seem to inhibit the final growth or impact the width of the anterior alveolar gap [13,17]. Early soft palate closure seems to allow for the faster improvement of the swallowing function and weight gain [17], which are relevant, particularly in countries where resources are limited. All these observations lead to the following conclusion: delaying the closure of the palate, i.e., later than 1.5 years of age, will favor the growth of the maxilla, but negatively impact speech development [12,15]. Although maxilla growth also remains a major concern for patients with clefts, speech intelligibility must be a priority when timing the surgeries [19,20]; orthodontics and surgery can correct the improper growth of the maxilla, but poor articulatory speech habits can be extremely difficult to eradicate after the age of five [21,22]. In this regard, the inverse Malek procedure, with the hard palate closed at ten months of age at the latest, can be performed within the advocated timing.

The treatment of a child with BCLP starts at the time they are diagnosed, ideally before birth, and ends when the child is fully grown. The concept of a multidisciplinary team is essential for a good and correct follow-up. The objective is to bring together specialists, i.e., pediatric plastic surgeons, maxillofacial surgeons, ear, nose, and throat (ENT) surgeons, speech therapists, nurses, and psychologists [5,9,23], to optimize the care and the functional and esthetic outcomes. This also requires the collaboration of obstetricians and geneticists.

This single-center retrospective cohort study focuses on the demographic and surgical data analysis of 30 patients affected by a BCLP and treated at the Children and Adolescent Surgery Department of the Centre Hospitalier Universitaire Vaudois (CHUV, University Hospital Lausanne, Switzerland) by the same surgical team.

## 2. Materials and Methods

### 2.1. Study Design and Patients

**Figure 1:**
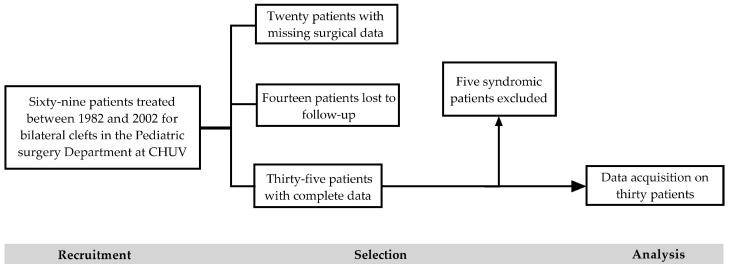
Patient selection process.

The present single-center retrospective cohort study analyzes data on 30 out of 69 patients born before 2003 with a BCLP. All were treated at the Children and Adolescent Surgery Department at CHUV by the same surgical team. The exclusion criteria were incomplete data due to follow-up being performed elsewhere and bilateral clefts in the context of a syndromic event (Figure 1). The present study was approved by the Cantonal Ethics Committee (i.e., CER-VD, BASEC-ID 2019-00468, 2019).

### 2.2. Data

#### 2.2.1. Collection, Protection, and Preservation

For this study, data were collected in physical form (i.e., paper) from medical archives or acquired using internal software at the CHUV (Siemens Soarian^®^, Version 4.5.100, Hillcrest Road, KC, USA, Archimede v3.5, and FileMaker v18 and v19) and reported in a password-protected Excel chart (v2019 and v2021, Microsoft Corporation, Redmond, WA, USA). The printed documents were kept under lock and key during the data acquisition period, and then returned to the archives or destroyed.

#### 2.2.2. Demographic Data

The demographic data acquired included gender, type of cleft (i.e., bilateral complete, bilateral complete/partial), antenatal diagnosis, and family history. Those data were expressed in frequency with percentages.

### 2.3. Organization

#### 2.3.1. Perinatal Period

Ultrasound antenatal diagnosis allowed us to anticipate and prepare information and material for the birth of the child. Prenatal discussions were organized to provide advice for the remaining course of the pregnancy. If the child was born with a cleft that was not previously diagnosed, a pediatric plastic surgeon assessed him/her within the first 24–48 h.

**Figure 2:**
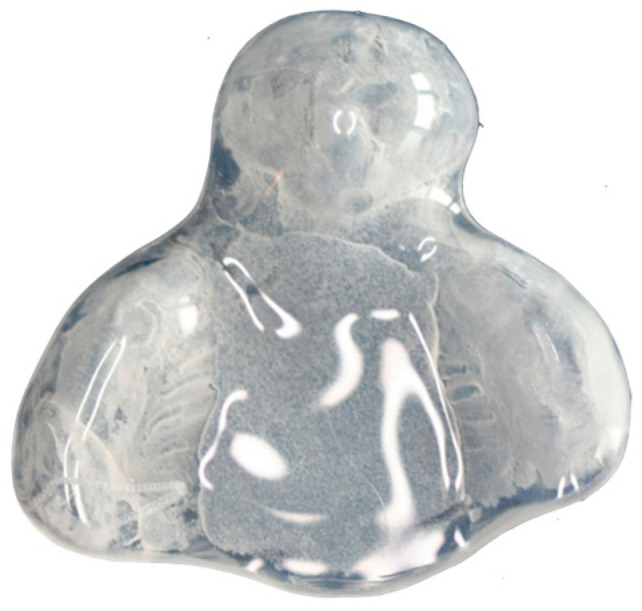
Palatal appliance.

The primary goal after the birth of a child with an open palate was to ensure correct feeding. A removable palatal appliance made of acrylic resin was built by the orthodontist soon after birth. The prosthesis had a rigid outer part and a softer more flexible inner layer that is in contact with the mucosa [23] (Figure 2). Then, a normal feeding bottle with a larger opening allowed the milk to run easily.

#### 2.3.2. Clinical Follow-Up Protocol

A close follow-up by a multidisciplinary specialized team was quickly organized (Table 1). The involvement of the parents in this global and complex care was considered essential. The aesthetic outcomes, language development, and the nature and schedule of the surgery were promptly discussed. All the children were assessed by a pediatric plastic surgeon (team leader) in the perioperative period, and then six months later and once a year up to the age of three and a half. Parents were provided with strategies to encourage babbling and early verbal communication when the child was one year old. At the age of three and a half, the child was evaluated by the multidisciplinary cleft team composed of the pediatric plastic surgeon, two pediatric ENT specialists, a craniofacial surgeon, an orthodontist, two speech therapists, and a psychologist. The child and its parents were then seen depending on the child’s needs, usually annually or biannually.

Psychological assistance for parents was provided from antenatal diagnosis until the child was 20 years old. Later, discussion sessions were organized for the children and their parents to provide the support needed if the social skills of the child/adolescent were affected, with resulting manifestations of anxiety, poor self-esteem, or depression.

#### 2.3.3. Speech

Speech assessment was performed at age three, and then every year if necessary. Perceptual speech evaluation by qualified therapists experienced in cleft pathology is the mainstay in our institution. To limit listener’s bias, the evaluations were made separately by two therapists, and then discussed and compared for all the patients. The children were interviewed in a quiet playroom in the presence of a parent. Standard upper airway assessments were documented, including the presence or absence of snoring, mouth breathing, apnea, and nasal airway obstruction. Articulation errors were divided into the following categories based on anatomical origin: labial, alveolar, palatine, velar, nasal, pharyngeal, and glottal. Backing, stops, and fricative sounds were recorded for each anatomical region, as were compensatory articulations (Table 2). Other articulation problems, such as the simplification, replacement, or deletion of consonants, were also recorded. Velopharyngeal insufficiency (VPI) or nasal air emission was evaluated according to the Borel-Maisonny classification (Table 3) [24,25]. Hyper-nasality, hypo-nasality, audible nasal emission, voice quality, misarticulations associated with VPI, and intelligibility were assessed. Nasal emission on separate phonemes was measured using a 622 Kay Electronics nasometer (Kay Elemetrics, Pine Brook, NJ, USA). Children with a type 1/2 Borel-Maisonny score or worse (i.e., 2, 2b, 2M, or 2/3) were referred to a speech therapist near their home and were seen by the University Hospital’s specialist once a year to evaluate their progress. Fluoroscopic velopharyngeal evaluations were not conducted. Clinical perceptual speech evaluations and nasometry were performed by the same two specialists before and after the operation.

#### 2.3.4. Hearing Assessment

Hearing evaluation was performed once a year by the ENT specialist, usually the same day as the speech assessment. The ENT specialist performed routine otoscopy, tympanometry, and checked for otitis media with effusion (OME). This was reported in a reproducible manner with impedance tympanograms using a Grason-Stadler GSI-28A tympanometer and total audiograms recorded with a Grason-Stadler GSI-16 earphone audiometer (Grason-Stadler, Littleton, MA, USA).

### 2.4. Description of Primary Surgeries

The surgical data were subdivided into plastic pediatric, ENT, and orthodontic/maxillofacial procedures. The data are expressed either as frequencies or in median with respective 25th and 75th quartiles (Q_25_–Q_75_) that include the middle 50% of the data.

#### 2.4.1. Surgical Timing

**Figure 3:**
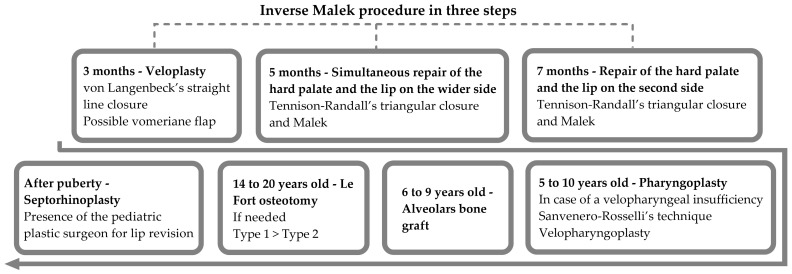
Surgical timing.

The inverse Malek procedure and the surgical timing are described in Figure 3.

#### 2.4.2. Pediatric Plastic Surgical Procedures

The Malek soft palate procedure was performed at three months according to von Langenbeck’s technique. The head of the child was placed on an adjustable support with neck hyperextension using a pad placed under the shoulders. The surgeon stood at the end of the table, with the assistant on his right and the anesthesiologist on his left. The patient’s eyes were protected with sterile strips and ocular ointment. The surgical equipment included the specific instruments required for palate surgery.

**Figure 4:**
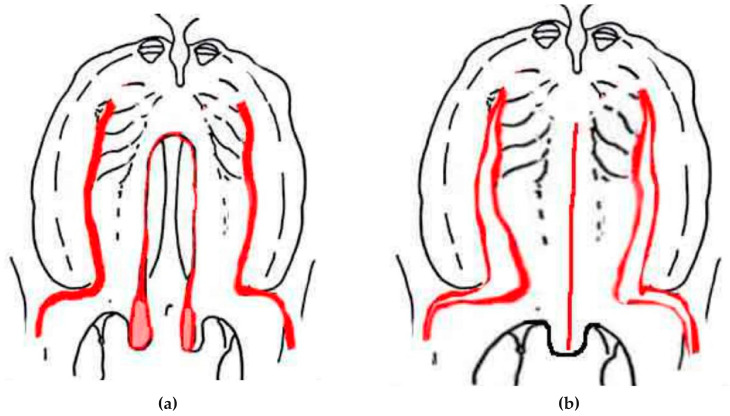
Von Langenbeck’s straight line closure. Planned incisions in red (**a**); flaps raised and sutured to midline (**b**).

First incisions were made along each margin of the cleft as far as the half-uvulae creating two flaps (Figure 4). Both hamuli were then broken with a Trélat elevator to relax the tendon of the tensor veli palatini muscle. Then, we proceeded to undermining, with the tissues being pushed back and carefully divided until the detachment of the soft palate was completed. For optimal flap medialization and wound tension, second lateral releasing incisions were placed medially to the greater palatine artery, thereby preserving blood supply to the palate. The mucosa that lines the nasal sides of the palatal shelves was easy to detach by displacing the velum towards the inside. This procedure created two layers, one in the nasal cavity and one in the oral cavity. The first layer of nasal mucosa was sutured with 5-0 or 4-0 absorber filament (Vicryl©, Ethicon Inc., Somerville, NJ, USA). When direct suturing was not possible, the vomer flap mucosa was used to reconstruct the nasal layer of the velum by making a longitudinal incision along the lower edge of the vomerine mucosa and nasal mucosa, on either side, from front to back, which was then sutured [26,27].

**Figure 5:**
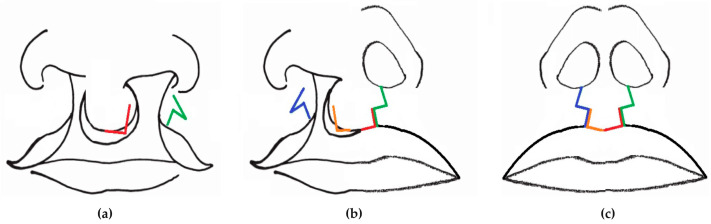
Bilateral cleft lip repair in two-stage by a Z plasty principle. First stage (**a**); second stage (**b**); result (**c**).

The aim of labioplasty at five months, and then seven months was to create two isosceles triangle flaps with incisions along both borders. This procedure was based on the principle of Z-plasty (Figure 5). The surgery began with mathematically accurate drawings of the lip repair. We then infiltrated a Bupivacaine hydrochlorate adrenaline solution to facilitate mucoperiostal undermining and to reduce bleeding through the temporary hemostatic effect of adrenaline. Additional length was gained between the two points thanks to the dissection of two triangular flaps that shared a common side. After the incision, the inversion of the flaps resulted in the inversion of the diagonals on the initially traced parallelogram. The long diagonal line replaced the short line, and vice versa, so that the desired additional length was obtained [26,27]. Skin without muscle was not used for the flap. The muscle layer on one side and the skin and underlying tissue of the prolabium (central part) were sutured together.

#### 2.4.3. ENT Surgical Procedures

**Figure 6:**
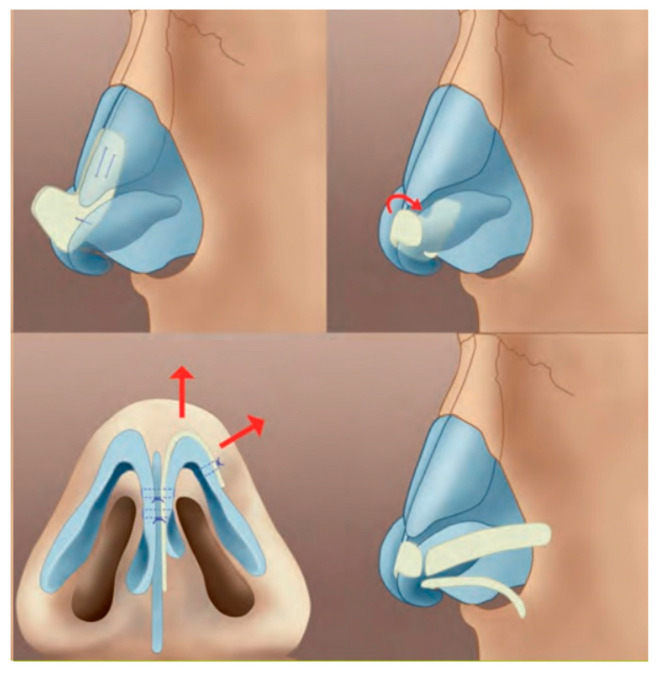
Septorhinoplasty. Tip and alar base symmetrization with cartilage grafts.

ENT interventions included the insertion of grommets and septorhinoplasty. Grommets were inserted in cases of OME visualized under a microscope after myringotomy. This procedure was performed at the time of the palatoplasty under anesthesia. Septorhinoplasty was planned at the end of the growth after the orthodontic treatment. The operation involved a small incision below the nose and necessitated a graft of cartilage taken from the ear [8] (Figure 6). The deviation of the nasal septum was also corrected during the same surgery. A thermoformable splint was used for several days or weeks. This surgery represented the final step after years and years of follow-up.

#### 2.4.4. Orthodontic and Maxillofacial Surgical Procedures

The indications for orthodontic treatment were both functional and esthetic. Malposition of the teeth was not usually treated because of the transitory nature of them. Parents were advised to control the exercise of the necessary dental hygiene. Our team believes that a long-lasting and possibly poorly effective conservative treatment must be avoided, not only for the sake of the child and their family, but also because of its cost [28]. The orthodontic treatment was correlated with the planning of the alveolar bone graft (ABG) when a maxillary cleft existed, with missing teeth (canines, incisors, and molars) or hypoplastic/improperly placed teeth [29,30]. The spongious bone for the graft was taken from inside a normal bone, usually from the iliac crest, or from the mandibular bone [31]. Around the age of twelve, we considered the relationship between the upper and lower jaws and the malposition of the teeth. An orthodontic approach was combined with maxillo-facial surgery if necessary.

In our institution, orthognathic surgery to correct facial disharmony is part of the process for children born with a BCLP. During planning, many factors, such as facial profile, intermaxillary discrepancies, and dento–alveolar relationship, are taken into account. An objective determination of the need for maxillofacial surgery was based from the analysis of lateral cephalograms. The anteroposterior relationship of the maxillary basal arch to the anterior cranial base was defined by the SNA, SNB, and ANB angles (S = sella; N = nasion; A = subspinal point; B = supramental point); anteroposterior jaw dysplasia was measured according to Wits appraisal (perpendiculars from points A and B onto the occlusal plane), and the distance from the upper lip to the e-line (line drawn from the tip of the nose to the chin) was the most used criteria. Maxillary advancement with Le Fort I osteotomy was the most common orthognathic procedure.

### 2.5. Description of Secondary Surgeries

**Figure 7:**
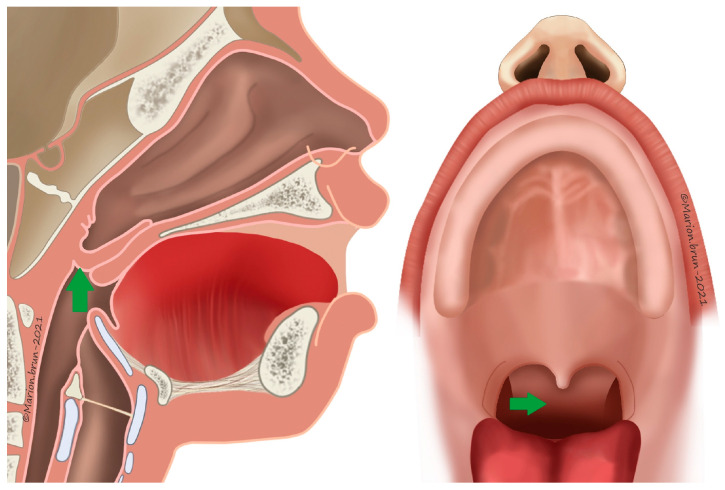
Cranial-based pharyngeal flap (green arrow).

The criteria for recommending pharyngeal flap surgery were based on perceptual analysis as follows: hypernasality, weak pressure consonants, weak pharyngeal musculature, and nasal emission (types 2M and 2/3 Borel-Maisonny). Cranial-based pharyngeal flaps (Figure 7) were used according to Schönborn and Sanvenero Rosselli [32,33]. A broad, cranially based pharyngeal flap was incised and elevated from the prevertebral fascia to be sutured to the nasal side of the incised velum. Knowing that the flap will shrink overtime, it was designed as large as possible, but we still left enough laxity for the direct closure of the donor site. The soft palate was dissected, and two mucosal flaps were prepared. These two flaps were incised on the dorsal velar side. The pharyngeal flap, including its muscle layer, was sutured to the nasal mucosa of the velum. In the midline, the entire thickness of two buccal flaps were joined and sutured to the surface of the flaps. Surgical success, established using speech considerations, was defined in terms of the elimination of perceptible hypernasality or oral resonance and instrumental evidence of complete velopharyngeal closure by nasoendoscopy. Velopharyngeal assessment was performed at six and twelve months after surgery. Surgical failure was defined in terms of persistent hypernasality and/or nasal turbulence during perceptual speech evaluation and of incomplete velopharyngeal closure evidenced by nasometry at least six months after surgery. Evaluation to determine the existence of sleep apnea was performed four months, and then one year after surgery (symptoms including fitful sleep, unresolved snoring, and daytime fatigue).

Secondary labioplasty in children with a BCLP is normally performed when the Cupid’s bow is not perfect or when the height of the repaired lip is too short. In the first case, surgery is simple, with a local correction and the alignment of white skin. In the second case, where the distance from the nose to the lip is not satisfactory, the height of the “normal lip” is calculated and compared with the opposite side.

### 2.6. Description of Complications

Oronasal fistulae (ONF) were closed if the children/parents expressed social discomfort due to oronasal regurgitation or in case of speech repercussion. The fistula was closed by the elevation of the mucoperiosteum of the entire palate. The simple closure of the hole was not performed. The closure of the fistula was postponed as long as possible to minimize adverse consequences on the growth of the maxilla.

### 2.7. Statistical Analysis

For this retrospective study of a limited case load, descriptive statistics and non-parametric tests were employed. Quantitative data were expressed either in frequencies with percentages, or in medians with measures of value spread. Qualitative data are described in frequencies with percentages. For simplicity, the percentages were rounded up to the nearest one decimal place, and a minor deviation of ±0.5% at most was deemed acceptable. The distribution of continuous variables was analyzed using a Mann–Whitney U test. A *p*-value < 0.05, two-tailed, was considered statistically significant. Calculations were performed using Excel (v2019 and v2021) and GraphPad Prism v. 8.0.2 (GraphPad Software, Inc., La Jolla, CA, USA).

## 3. Results

### 3.1. Study Design, Patients, and Data

A total of 30 out of 69 patients with a BCLP met our selection criteria for further analysis (Figure 1). Twenty-three were males, and seven were females (Table 4). Their years of birth ranged from 1982 to 2002. The number of patients with a BCLP per year varied from one to five. An increase in cases was seen from 1990 on.

The clefts were complete in the majority of the total population (80%). Partial clefts were seen only in the male population. Four boys had a partial left cleft. One male child had a partial right cleft, and one other had a bilateral partial cleft. Antenatal diagnosis was performed for three out of thirty patients (10%). A positive family history was found in six children (20%) (Table 4).

### 3.2. Primary Surgeries

**Figure 8:**
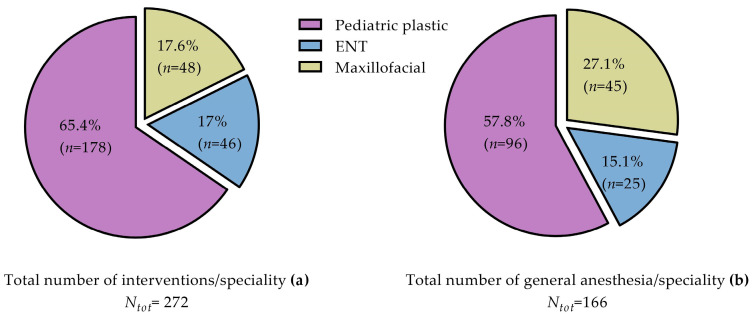
Pie charts showing percentage and absolute number of interventions (**a**) and GA cases (**b**) per surgical field for the 30 patients considered.

Interventions for the 30 evaluated patients were divided according to the surgical specialties (Figure 8). In total, 272 operations were performed; all the surgical fields were considered (Figure 8a). The median numbers of interventions and general anesthesia (GA) cases out of all the surgical fields concerned were 9 (7–11) and 5.5 (4.25–6) (Figure 8a,b). The number of GA cases was also evaluated according to the different specialties. Labial repairs, palatal fistula treatments, and autologous fat transfers were almost always combined with another surgery (i.e., ENT or maxillofacial). Thus, total number of interventions is higher because several procedures were performed under GA. If a premaxillary osteotomy was necessary, it was performed systematically with the ABG. GA for pediatric plastic surgeries was the most common (57.8%). The proportion of cases of GA use for maxillofacial procedures was 27.1%, and this was 15.1% for the ENT interventions (Figure 8).

**Figure 9:**
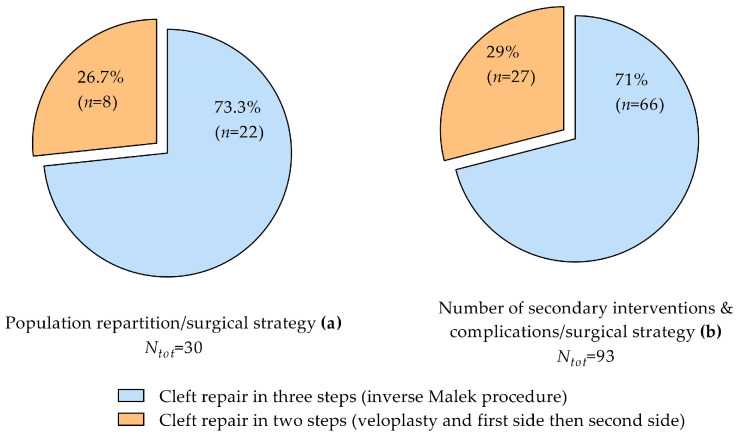
Pie charts showing groups according to the surgical strategy undertaken for BCLP repair (**a**) and their respective number of secondary interventions and complications (**b**). Numbers are expressed as % and absolute values. Secondary interventions: pharyngoplasty and lip revisions; complications: oronasal fistulae.

The vast majority of the population (73.3%) underwent the inverse Malek protocol for cleft closure in three steps. Eight children (26.7%) had a two-step closure, including veloplasty, with the hard palate and lip on one side (the most affected), and then the hard palate and lip on the second side (Figure 9a).

The median age for veloplasty was 3 (3–4) months. The first side closure of the hard palate and the lip was performed at a median age of 6 (5.25–6.75) months, and the second side closure was performed at 8.5 (8–9) (Table 5).

Grommet insertion was needed for 56.7% of the total population. Thirteen patients out of seventeen had one intervention, and four had two. The median number of interventions per patient was one (0–1) (Table 5). Eighteen patients out of thirty (60%) developed OME, and in eleven of them (36.7% of the total population), it was performed in combination with acute otitis media (AOM). Grommets were inserted in 16 of them. Three children out of thirty (10%) developed AOM alone without the need for myringotomy. One of these children eventually required a hearing aid. Eight patients out of the total population (26.7%) had no hearing issues.

Tonsillectomy and adenoidectomy were performed on five patients out of thirty (16.7%). Tonsillectomy alone was performed on one patient. Three children had adenoidectomy alone, and one had both tissues removed. The ages of intervention are detailed in Table 5.

Septorhinoplasty was performed in 53.3% (16/30) of the cases. Of these sixteen patients, three needed a revision rhinoplasty. The median age for the primary procedure was 204 months (162–243) (Table 5).

Twenty-five patients out of thirty (83.3%) had an ABG. Premaxillary osteotomy was performed concomitantly in three patients. The median age of the patients undergoing the operation was 120 (108–120) months (Table 5). Sixteen children out of the total population (53.3%) had Le Fort osteotomy. Type I was used in sixteen cases, and type II was used in two. The median age for these interventions was 216 (204–216) months (Table 5).

### 3.3. Secondary Surgeries

No statistical significance in the variance in medians was found when comparing the different strategies with the occurrence of secondary surgeries or complications (Mann–Whitney U = 85.5, N1 = 22, N2 = 8, *p* = 0.901 two-tailed, Figure 9b). Only one child treated with the inverse Malek protocol had zero complications.

Pharyngoplasty was performed on 11 patients (36.7%). Two out of eleven had to undergo a second surgery. The median age was 96 (72–120) months (Table 5).

**Figure 10:**
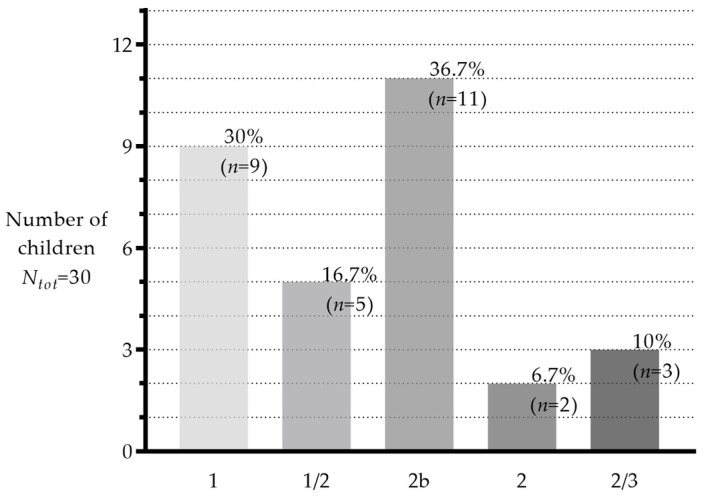
Classification by columns of the 30 patients according to Madame Borel-Maisonny at the end of speech therapy. X axis, final types; Y axis, number of patients.

The median age of phoniatrics onset was 3.4 (3–3) years. Half of the children showed good-to-excellent results at the final evaluation. Nine patients (30%) had good phonation without any nasal air leak during articulation (classified as one on the rating scale). Five (16.7%) had only occasional nasal air leaks (classified as ½). Eleven patients (36.7%) had a constant nasal air leak, which was not audible (classified as 2b). Two children (6.7%) had a permanent nasal air leak, and three (10%) developed articulatory compensation phenomena (classified as 2 and 2/3, respectively). No child was classified as 2M (Figure 10).

Under lip corrections, we include all the procedures linked to the primary palate, and also all the revisions of the subunits of the lip. Twenty-three out of thirty (76.7%) had lip revisions. Five had one surgery, fifteen had two, two had three, and one needed four interventions. The median number of interventions per patient was two (1–2) (Table 2).

### 3.4. Complications

Palatal fistulae were the major complications in 21 (70%) patients and required one, two, or three repairs. Respective median ages (months) for reparation were 84 months (60–120) for one repair (11 patients), 156 (114–177) for two repairs (6 patients), and 210 (201–222) for three repairs (4 patients). Out of these twenty-one patients, eleven required one single repair, six had a second intervention, and four had a third (Table 5).

Three children had maxillofacial complications, including insufficiency of the alveolar graft, necrosis of the graft, and acute sinusitis.

## 4. Discussion

This single-center retrospective cohort study considers the long-term results of our institutional specific surgical protocols for the treatment of children with a BCLP. All the children discussed were treated and followed-up exclusively at the Children and Adolescent Surgery Department of the CHUV by the same surgical team. This shows that the inverse Malek procedure is related to a high risk of fistula, but has good long-term speech results.

Our study gives data on bilateral clefts, which are, for the most part, complete and symmetrical. In our center, Malek’s protocol for bilateral cases is applied in the following three steps: a soft palate procedure at three months, a hard palate procedure and labioplasty on the first side at six months, and then second labioplasty on the other side and eventual palatal adjustment, if needed, at eight months. But, as described in our results, the surgery differed in eight cases, i.e., closure in two steps. The timing of the surgery as described previously should always be observed to obtain a satisfactory balance between speech intelligibility, deglutition, and breathing and to preserve the normal growth potential of the affected structures. Different surgical models and timings have been exploited since 1970 [12]. A fine balance exists between delaying the closure of the palate (both hard and soft) and creating scars on the periosteum. The first point can result in speech problems [14], and the second can induce growth abnormalities of the midface [12,13]. We know that the timing of soft tissue and palatal closure is more important than their sequence [13,15,16,17,18], and delaying hard palate closure results in speech impairment, without showing any benefit for maxillofacial growth [14,19,20]. Furthermore, the early closure of the soft palate may reduce the width of the hard palate cleft, but does not seem to inhibit the final growth or impact the width of the anterior alveolar gap [13,17]. If it is true that early soft palate closure (i.e., Malek’s protocol at 3 months) allows for the faster improvement of the swallowing function [17], it does not seem to give a particular advantage to the other surgical protocols. That being said, the main point that supports all these different protocols is that delaying the closure of the palate will favor the growth of the maxilla, but negatively impact speech development [12,15], and although maxilla growth is a major concern for patients with clefts, speech intelligibility must be a priority when timing the surgeries [19,20]. Orthodontics and orthognathic surgeries can correct the improper growth of the maxilla, but poor articulatory speech habits are extremely difficult to eradicate after the age of five [21,22]. Malek’s procedure was performed at several stages in order to reduce the risk of necrosis due to the extensive denuding of tissue and to adapt surgical planning according to the healing and growth of the child. Since then, using a superior technique, the adequate use and mobilization of the tissue, as well as multilayered approximation of the structures, several authors [10,11,34] depicted successful one-stage repair for children with a BCLP, and they generally addressed the nose during the cleft repair.

The results of the present study guided the adjustment that we made in 2014. To reduce the number of oro-nasal fistulae, we altered the timing of operations, which are the closure of the total cleft at four months on one side, and at six months on the other side.

In the presented paper, the rates of complications were similar for all our surgical protocols and not always comparable to what is found in the literature [34,35]. Reports about later outcomes in cases with BCLPs are very rare. This is probably partly linked to the small size of the sample and to the difficulty for one specific surgeon or one surgical team to follow all the patients with a BCLP until completion and the stabilization of skeletal and soft tissue growth [34]. Chouairi et al. [35] compared many peri-operative complications of unilateral and bilateral cleft lips and, when conducting the propensity matching of 855 cases for each group, found no significant differences in terms of hospital stay, death, surgical site infection, sepsis, bleeding, organ (liver, kidney, and heart) malfunction, readmission, or reoperation. Unfortunately, the history of lip revision and its long-term outcomes, such as presence of fistulae or the need for alveolar bone grafting, could not be derived from their database.

The current literature reports a variable incidence rate of ONF, ranging from 4 to 60% [36,37,38,39,40,41,42,43], depending on the tissue quality of the patient and on surgical skill and the type of cleft. Children with a BCLP are known to have a high incidence of palatal fistulae [40,41,42], and they remain a major complication in the early primary palate technique. Their first consequence is the leakage of fluids or soft food through the nose. It is known as the “signe du chocolat” when ingested chocolate falls from the nostril. The second consequence is a speech disorder called rhinolia, with nasal air emission during speech. The indication for repair of a fistula depends on the severity of its manifestation, but the child must be followed by a speech pathologist to make sure that compensatory phenomena due to the escape of air are not present (Table 2).

Some authors advocate that localization and the technique used do not appear to play a preponderant role in the occurrence of a fistula [37,38,39,40,41,42]. Nevertheless, Sommerland’s technique, which consists essentially in a modified preservation technique of von Langenbeck with the closure of the whole palate at six months, reported an occurrence of 35% ONF in 28 patients with a BCLP [44]. Lateral relaxing incisions were necessary in 70% of bilateral cleft cases, against 20% in the unilateral cases, and seemed to play a major role in fistula occurrence. Furlow’s double-opposing Z-plasty technique, which also closes the soft and hard palates appears to caused fewer ONF (up to 10%) than von Langenbeck’s technique [45,46]. However, lateral incisions are nonexistent in Furlow’s technique, and its application is difficult in treating wider clefts [47]. These studies give some perspectives on our take of palatal repair, and as mentioned before, since 2014, we have been operating our children at four months on one side and at six months on the contro-lateral side, with the minimal dissection of the hard palate.

Regarding the current study, despite the early closure of the velum, we found a very high incidence of palatal fistulae (70%), which required ulterior repairs in approximately half of the children. For closure, we elevated the mucoperiosteum of the entire palate. This surgery was postponed as long as possible to minimize adverse consequences on the growth of the maxilla.

We also found to more VPI that required a pharyngeal flap compared to the other techniques [44,48], but the literature offers scant information concerning the need for pharyngoplasty in cases with a bilateral cleft [49,50,51]. The percentages vary from 16 to 38% when all types of clefts are considered. Pharyngoplasty is required when primary repair surgery results in insufficient velopharyngeal function. The surgical procedures include lengthening of the palate [23,52] (pushback palatoplasty, Furlow double-opposing z-plasty, and pharyngeal flap, with the latter commonly used in our center since 2014), sphincter pharyngoplasty, and the enlargement of the posterior pharyngeal wall with injectable materials (autologous fat or hyaluronic acid) [53,54,55]. Pharyngeal flaps can be based superiorly, inferiorly, or laterally [56]. We mainly practice the superiorly based flap procedure, as it has proved efficient and very successful [57,58]. The failure of the procedure is certain when hypernasality persists. In the literature, the rate of failure is estimated at up to 20% [57,59,60].

Children with a BLCP are subject to a delayed speech development due to the palatal cleft. Assessments can be performed either subjectively, by a speech therapist, or using pressure-flow technology measurement [23,61]. In our center, as in many other French-speaking institutions [25], we evaluate speech or nasal air emission based on the Borel-Maisonny classification. Although this was not performed in the population studied in this paper, in 1999, we introduced video nasopharyngeal endoscopy (VNPE) as a systematic pre-surgery work-up; this allows for the direct observation of velopharyngeal movement during speech [23]. The cooperation of the child is a prerequisite, and the interpretation can be operator-dependent. In our center, speech therapy is initiated at the age of about three years, but exercises to strengthen the velopharyngeal muscles can be performed by children as young as 12 months [23]. The parents must be made aware of their role in early speech acquisition and help the children to improve breath control and correctly position the tongue and lips. Speech therapy alone is insufficient, and regular daily exercises at home are an integral part of the treatment [62]. In 2002, we introduced speech therapy workshops for parents and their children (two-hour sessions twice a year with four or five patients of the same age and presenting the same cleft type), where the speech therapist shows the parents how to work with their children at home (guidance). This aims at strengthening the velopharyngeal muscle complex. Therapy sessions can only do so much, and regular daily exercises at home must be performed to complete the treatment. Babbling and strategies to stimulate facial muscle activity and improve their flexibility are encouraged.

From an ENT point of view, infants with clefts can more easily develop OME because of the pathological cranial morphology that compromises the function of the tensor veli palatini muscle on the Eustachian tube’s dilatation mechanism [63]. Muntz [64] notes that whereas approximately 70% of all healthy children will have suffered one or more episodes of OM by the age of three, 90% of children with a CP will. This number varies between ethnic groups, with Chinese children being the least affected. These patients will need long- or short-lasting grommets, depending on their condition, to prevent hearing problems. Berryhill [65] describes a hearing loss from −25 to −40 dB in cases of OME, and myringotomy is often a necessity. The meticulous examination of the middle ear is crucial, and tympanometry has been the most common and widely accepted tool for assessing the presence of fluid in the middle ear [66,67,68]. The use of grommets is largely debated because both their indication and the correct time to insert them are not clearly defined. The complications caused by their insertion are numerous, for instance, atelectasis, perforation, tympanic membrane scarring (tympanosclerosis), and cholesteatoma [67,68]. The pediatricians who treated the children in our study were instructed in the special care needed to diagnose chronic serous otitis media and were asked to perform routine otoscopy and check for OME. However, this instruction was not effectively followed, as described in a previous publication [69]. Every child is now checked with a microscope by an ENT during our first operation. If needed, both the grommets are placed simultaneously.

Other studies have shown that there is no difference in dental anomalies between unilateral and bilateral clefts [35,70]. Early bone grafting seems to impair maxillofacial growth [71,72] and is therefore rarely used [36]. In our center, we practice alveolar bone grafts at around ten years of age, and if required, we stabilize the premaxilla during the same intervention. As described by Padwa and Mulliken [36], numerous studies have shown that an ABG at the age of mixed dentition is not deleterious to facial growth. The rate of success is usually high (more than 90%), but it decreases with age (beyond 12 years of age) [73,74].

Nasal deformities in patients with BCLP include the following features: the tip of the nose presents a shorter medial crus and a longer lateral crus of the lower lateral cartilage on both sides; the columella is short, with a wide base; the nostrils have a horizontal orientation; the alar bases are mispositioned in a latero-inferior conformation; and the nasal floor is absent. The septum is usually in the midline, but some deviation can occur on the less-affected side when the cleft is incomplete on one side [75,76]. When primary rhinoplasty is insufficient, open rhinoplasty and/or septal correction may be necessary. In a series of 800 patients (186 with a UCL, 57 with a BCL, 473 with a UCLP, and 84 with a BCLP) treated over 18 years, Farouk [75] reported the need for secondary open rhinoplasty in 258 patients (32.25%). Among these 258 cases, 30 patients had a BCLP, corresponding to 11.6% of this subcategory. In our center, the pediatric plastic surgeon performs the primary repair of the nose, but thanks to our multidisciplinary approach, an ENT specialist from the cleft team intervened later in the patient’s life, between 13 and 20 years of age, to correct eventual functional or aesthetical problems; this may explain our higher number of secondary open rhinoplasties (56%).

Lip corrections were necessary for 79% of our population, and more than half of those required a second surgery. The percentage mentioned considers all the esthetic subunits of the lips, meaning not only the lip per se, but also lateral segments and philtral column. Mulliken et al. [34] reported that for 50 children with a BCLP, a revision rate of 33% was necessary for those with a complete cleft, and of 12.5% for those with an intact secondary palate. Secondary revision was rarely necessary. Most revisions included sulcoplasty and the adjustment of the median raphe, and fewer revisions included columellar narrowing or work around the nasal base. The number of patients with a BCLP with an intact secondary palate (usually up to 10% in complete bilateral cleft) was not specified. Rossell-Perry [77] described nasoalveolar molding as a good alternative for the primary simultaneous repair of the lips and nose in those with a BCLP. The concept of simultaneous cheilorhinoplasty was underlined by Mulliken and Millard [78,79]. They observed that bilateral labial closure without addressing the nose accentuated the nasal deformity. This finding introduced a fundamental change in the modern surgical strategies [80,81,82]. The general benefits of molding are the improvement of nasal appearance, the stability of the nasolabial esthetic, less scar tissue, the restitution of a maxillary arch with the (sub-)closure of the alveolar gap, and less need for revision surgeries, which have demonstrated advantages of pleasing and stable nasolabial esthetics with less scar tissue, an intact maxillary dental arch without any oronasal fistulas, and a reduction in the number of surgical revisions [77,83]. However, concrete conclusions about the benefits of nasoalveolar molding need to be made in future studies [77], and the application of such devices demands the specific expertise of surgeons and orthodontists [83].

Patients with a cleft undergo many interventions during childhood and early adulthood. McIntyre et al. [84] conducted a retrospective study to identify the clinical features that may contribute to the number of procedures and anesthesia complications suffered by children affected by an orofacial cleft. Within a sample of 71 patients, they found that patients with a BCLP underwent, on average, 10 procedures against 5.3 and 5.9 for isolated CLs and CPs, respectively (*p* = 0.01). They also emphasized that patients treated by multiple plastic pediatric surgeons required more surgical interventions. This confirms the importance of a continuity of care by the same team. Race and sex did not significantly influence the number of surgeries.

The retrospective nature of this paper is obviously a major limitation, and its monocentric aspect prevents us from generalizing our results. Also, when selecting our study population, we chose to be as thorough as possible concerning the inclusion criteria, thereby limiting our data collection. It was important to us to be coherent regarding our inclusion and exclusion criteria and the identification of specific issues in the care of patients with a bilateral cleft. Unfortunately, and as already stated above, the literature lacks comparisons in this field, thereby limiting the interpretation of our results. When talking specifically about the items analyzed, we could have been more precise concerning various secondary repairs, especially in the cases of lip flaws and palatal fistulae. Our paper only reports quantitative data and lacks qualitative descriptions; for instance, palatal fistulae were not described according to their localization, and lip revisions comprised all the interventions, no matter the type. Nevertheless, we believe that our data can provide some useful information about past surgical strategies and techniques from which we can learn to progress and to improve the care of patients with bilateral clefts. Finally, and despite conducting a close follow-up up to the age of 20, our study is sadly short on information about social integration and individual psychosocial development during this complex and long period, even though these are the ultimate goal of cleft surgery.

## 5. Conclusions

The management of a cleft starts at the time it is diagnosed, ideally before birth, and ends when the child is fully grown. Out of a total of 69 patients with a BCLP, we carefully selected 30 patients for further analysis. Our surgical approaches and the age of the patients at the time of the surgical interventions seem consistent with what other cleft centers report. Although we were not able to prove a low complication rate or the advantage of a specific technique, we remained confident in our standard of care at the CHUV. Our study brings precise information about children with BCLPs, for whom operative techniques are particularly challenging, and calls for improvement in the surgical field. Future studies centered on patients with BCLP will allow for proper data comparisons to optimize the procedures and prevent complications. A multi-center approach or a prospective study design will help to validate and expand our findings. At this stage, complications seem inherent in cleft surgery, as shown in this article and the vast literature on the subject. We hope that our paper will contribute to the efficacy of the management of this multidisciplinary disorder, from birth to adulthood.

## Figures and Tables

**Table 1:** General follow-up of children with bilateral cleft.

When?	What?	Who?
Antenatal	Information and preparation Psychological help Genetic counseling	Pediatric surgeon Psychologist Geneticist (possibly)
Birth	Information Alimentary issues	Pediatric surgeon/Nurses Orthodontist
2–3 months	Veloplasty	Pediatric surgeon
3–6 months	Palato- and labioplasty (1st side)	Pediatric surgeon
5–10 months	Palato- and labioplasty (2nd side)	Pediatric surgeon
Through the first years of life	Check-up Parental guidance Grommet’s insertion Tonsillectomy/adenoidectomy	Pediatric surgeon Pediatric surgeon ENT specialist ENT specialist
3 years	Appraisal Speech therapy	Team Speech therapist
7–9 years	Follow-up Orthodontic evaluation	Team Orthodontist
7–20 years	Follow-up Pharyngoplasty Secondary surgeries: fistulae, lip correction ABG/Osteotomies Septorhinoplasty	Team Pediatric surgeon Pediatric surgeon Maxillofacial surgeon ENT specialist
18–20 years	Genetic counseling	Team

ENT, ear, nose, and throat (otolaryngology) specialist; ABG, alveolar bone graft.

**Table 2:** Sounds and signs recorded.

Misarticulations not related to VPI	Oro-nasal, stigmatism, posteriorisation, deletion of consonants, confusion, fricative sounds, and backing
	Heavy misarticulation: articulation compensations, glottic sounds, and raucity
Related to VPI	Voice trouble: hypo-/hyper-nasality and raucity
	Facial compensatory movements: synkinesis
	Added sounds: snoring, mouth breathing, and clicks

VPI, velopharyngeal insufficiency.

**Table 3:** Borel-Maisonny classification.

Type 0	No phonation.
Type 1	Excellent phonation, no nasal air emission.
Type 1/2	Good phonation, intermittent nasal air emission, good intelligibility.
Type 2b	Phonation with continuous nasal emission, good intelligibility, no social discomfort.
Type 2	Phonation with continuous nasal emission.
Type 2M	Phonation with continuous nasal emission, bad intelligibility.
Type 2/3	Phonation with continuous nasal emission with compensatory articulation, bad intelligibility.

**Table 4:** Demographic data of the studied population.

	Female *N* = 7/30 (23.3%)	Male *N* = 23/30 (76.7%)	Total Population *N* = 30 (100%)
BCLP *n* (%)			
Complete (both sides)	7/7 (100%)	17/23 (73.9%)	24/30 (80%)
Partial (left)	0	4/23 (17.4%)	4/30 (13.3%)
Partial (right)	0	1/23 (4.2%)	1/30 (3.3%)
Partial (both sides)	0	1/23 (4.2%)	1/30 (3.3%)
Antenatal diagnosis *n* (%)	1/7 (14.3%)	2/23 (8.7%)	3/30 (10%)
Positive family history *n* (%)	2/7 (28.6%)	4/23 (17.4%)	6/30 (20%)
From maternal side	1/2 (50%)	1/4 (25%)	2/6 (33.3%)
From paternal side	1/2 (50%)	2/4 (50%)	3/6 (50%)
Sibling	-	1/4 (25%)	1/6 (16.7%)

*N* and *n*, number in absolute; BLCP, bilateral cleft lip and palate. For clarity, percentages were rounded off to the nearest one decimal place, and a minor deviation of 0.5% at most was accepted when summing up the numbers.

**Table 5:** Number of patients and median ages at interventions according to surgical field.

	*N* (%) of Patients/Intervention	Median (Q_25_–Q_75_) Ages (Months) ^1^
Interventions by the pediatric surgeon		
Veloplasty	30/30 (100%)	3 (3–4)
Hard palate closure and labioplasty	30/30 (100%)	
First side		6 (5.25–6.75)
Second side		8.5 (8–9)
Delta		3 (2–3)
Palatal fistulae	21/30 (70%)	
One single repair	11/21	84 (60–120)
Need of second repair	6/21	156 (114–177)
Need of third repair	4/21	210 (201–222)
Lip corrections	23/30 (76.7%)	2 (1–2) ^1^
One time	5/23	
Two times	15/23	
Three times	2/23	
Four times	1/23	
Pharyngoplasty	11/30 (36.7%)	
One single intervention	9/11	96 (72–120)
Need of second intervention	2/11	At 118 and 124 months
Interventions by the ENT specialist		
Grommet’s insertions	17/30 (56.7%)	1 (0–1) ^1^
One time	13/17	
Two times	4/17	
Tonsillectomy and adenoidectomy	5/30 (16.7%)	
Tonsillectomy alone	1/5	At 48 months
Adenoidectomy alone	3/5	At 12, 24 and 60 months
Both	1/5	At 84 months
Septo(rhino)plasty	16/30 (53.3%)	204 (162–243)
Need of revision	3/16	At 228, 228 and 240 months
Interventions by the maxillofacial surgeon		
Alveolar graft	25/30 (83.3%)	120 (108–120)
With premaxilla osteotomy	3/25	
Le Fort osteotomy	16/30 (53.3%)	216 (204–216)
Type I	14/16	
Type II	2/16	

*N* (%), numbers in absolute and respective percentages. For logical reasons and clarity of presentation, % was not noted for children who had several revisions of the same procedure throughout follow-up. ^1^ For lip corrections and grommets insertions, age of intervention was not collected during prior material analysis. Respective medians express number of interventions/patient, and calculations were performed on the 30 patients considered to obtain operative data.

## Data Availability

The study was retrospective in nature. No new data were created, and only archival data were used for description and analysis. Raw data are unavailable due to privacy and ethical restrictions.
